# Food Insecurity and Mental Health Among High School Students: Evidence From National and Virginia Youth Risk Behavior Surveillance Data

**DOI:** 10.1111/josh.70172

**Published:** 2026-06-08

**Authors:** Nigel James, Asef Raiyan Hoque, Jada Pedersen, Huong Doan, Liam Hayes, Leah Suggs

**Affiliations:** ^1^ Department of Health Studies University of Richmond Richmond Virginia USA; ^2^ Covenant HealthCare College of Medicine Central Michigan University Mount Pleasant Michigan USA

**Keywords:** adolescent mental health, food insecurity, health equity, nutrition, school health, YRBSS

## Abstract

**Background:**

Youth mental health problems are rising nationally. Virginia ranks among the lowest states in youth mental health access and has concentrated pockets of food insecurity (FI), underscoring the urgency of policy responses. FI is a known risk factor, yet surveillance often relies on single‐item measures. We introduce a multidimensional Food Insecurity Index (FII) to examine associations with adolescent mental health.

**Methods:**

We analyzed 2023 Youth Risk Behavior Surveillance System data from Virginia (*n* = 1866) and the United States (*n* = 10,724). The FII combined five indicators capturing acute scarcity and chronic dietary compromise. Survey‐weighted logistic regression estimated adjusted odds ratios (AORs) for persistent sadness or hopelessness.

**Results:**

Each 1‐point increase in FII was associated with higher odds of persistent sadness or hopelessness in Virginia (AOR = 1.066, 95% CI: 1.005–1.130) and nationally (AOR = 1.087, 95% CI: 1.062–1.112). Female students had higher odds than males, and bullying was a strong correlate. A dose–response pattern was observed.

**Implications for School Health Policy, Practice, and Equity:**

Integrating FI screening with nutrition and behavioral health supports may help identify and address overlapping risks.

**Conclusions:**

Multidimensional FI is consistently associated with poorer mental health among high school students.

## 1 Introduction

Adolescent mental health in the United States is in crisis [1, 2]. The Centers for Disease Control and Prevention (CDC) reports that about two in five high school students experienced persistent feelings of sadness or hopelessness in the past year, and roughly one in five seriously considered suicide [3]. These trends have worsened over the past decade and reflect multiple, intersecting stressors, including socioeconomic instability, social exclusion, discrimination, academic pressures, and the pervasive impact of digital media [4, 5].

In Virginia, the crisis is compounded by structural barriers to care. The state ranks 48th nationally in youth mental‐health service accessibility. Several structural factors contribute to Virginia's low ranking in youth mental‐health service access. Workforce shortages remain a persistent challenge, particularly in rural regions that the Health Resources and Services Administration (HRSA) has designated as mental health (Health Professional Shortage Areas [HPSAs]) [6, 7]. In addition, behavioral‐health providers are unevenly distributed across the state, with most specialists concentrated in urban centers, leaving many communities with limited or no access to child and adolescent psychiatrists [8]. Insurance coverage gaps, long wait times for appointments, and limited integration of behavioral health services within school systems further constrain access to timely care [9, 10]. Together, these structural barriers contribute to a landscape in which demand for adolescent mental‐health services is rising while the capacity of the system to respond remains limited.

County‐level signals in Henrico underscore these pressures: over the past 3 years, Henrico County Public Schools documented a 42% increase in suicide‐risk screenings; in 2024, the county's Mental Health & Developmental Services reported approximately a 30% rise in youth requesting same‐day access to services. While the Henrico CARES initiative seeks to expand prevention, school‐based care, and care navigation, timely access remains a challenge [11].

Virginia also faces material hardship that intensifies psychological risk. The statewide food‐insecurity rate was 8.1% in 2021, below national levels, but averages mask sharp within‐state disparities tied to food deserts and economic disadvantage [12]. Nationally, food insecurity (FI) reached 13.5% in 2022, situating Virginia's local pockets of high need within a broader rise.

FI—limited or uncertain access to adequate food—is increasingly recognized as an important social determinant of adolescent mental health [13, 14, 15, 16, 17, 18, 19, 20, 21, 22, 23]. Beyond physiological consequences, FI can affect adolescent mental health through several interconnected pathways. Uncertainty about access to food generates chronic psychological stress within households, which may heighten feelings of anxiety, sadness, and hopelessness among youth [24, 25]. Adolescents in food‐insecure households may also experience social stigma, embarrassment, and social withdrawal related to material hardship, which can weaken peer relationships and reduce engagement in school activities [26, 27]. Nutritional compromise due to limited access to balanced diets may further affect cognitive functioning and mood regulation. In addition, FI often co‐occurs with broader socioeconomic adversity—including housing instability, parental financial stress, and neighborhood disadvantage—that can compound emotional distress [28, 29, 30]. These pathways are increasingly supported by empirical evidence linking FI to adverse mental health outcomes among adolescents. Using pooled 2017 Youth Risk Behavior Surveillance System (YRBSS) data from 11 states, Brown and colleagues found that food‐insecure high school students were nearly twice as likely to report persistent sadness or hopelessness and more than three times as likely to attempt suicide. Their analysis—based on the optional YRBSS single‐item hunger measure—provides a practical multistate benchmark for understanding this association among US adolescents [17]. However, despite growing national evidence, relatively little research has examined how this relationship manifests in specific state contexts such as Virginia, where both FI and mental health service constraints present important policy challenges.

This study uses Virginia—a state with limited youth mental‐health service capacity and concentrated pockets of FI—as a policy‐relevant context to assess whether patterns observed nationally are similarly present within a high‐need state setting.

A persistent challenge in state‐specific analyses is variation in the inclusion of direct food‐insecurity items across YRBSS administrations. In Virginia, the YRBSS does not include the hunger item; to enable state–national comparisons despite this absence, we developed a composite Food Insecurity Index (FII) from routine diet items [31]. Grounded in established behavioral and nutritional frameworks, the FII integrates indicators of acute scarcity (e.g., breakfast skipping) and chronic dietary compromise (e.g., low fruit and vegetable intake, high sugar‐sweetened beverage consumption, low water intake). Details of FII construction are provided in the Methods. This approach enables Virginia‐specific estimates and direct comparisons with national patterns using a common measure, positioning schools not only as surveillance sites for health risks but also as scaffolds for resilience amid material insecurity.

We aim to (1) estimate the association between FI and mental‐health outcomes among Virginia and US high school students using a novel FII; (2) compare patterns between state and national samples; and (3) discuss implications for school‐based screening, intervention, and prevention strategies. By integrating a theory‐driven measure into a well‐established surveillance system, this study extends prior research and provides actionable evidence for educators, school‐health professionals, and policymakers addressing the intertwined challenges of FI and adolescent mental health.

## 2 Methods

### 2.1 Study Design and Data Source

This cross‐sectional study used data from the 2023 YRBSS, a school‐based survey administered biennially by the CDC. The YRBSS includes both a nationally representative survey conducted by the CDC and separate state‐level surveys administered by state and local education and health agencies (CDC) [31, 32].

The national YRBSS employs a three‐stage cluster sample design to obtain representative samples of students in Grades 9–12 attending public and private high schools [32]. Surveys are administered anonymously during a regular class period and completed voluntarily by students.

In addition to the national survey, state‐level surveys aligned with the YRBSS framework are conducted independently by state agencies using jurisdiction‐specific sampling strategies and questionnaires. These state‐level surveys are not subsets of the national sample [31]. In Virginia, the survey is administered biennially in randomly selected public high schools through a collaboration between the Virginia Department of Health, the Virginia Foundation for Healthy Youth, and the Virginia Department of Education [33].

Consistent with prior research using state‐level YRBSS data, these surveys typically employ a two‐stage cluster sampling approach to obtain representative samples of high school students, with sampling weights applied to account for nonresponse and demographic distributions [34].

This study used both the national YRBSS dataset and state‐level YRBSS data from Virginia. For descriptive analyses, participants with missing data on the exposure or outcome were excluded. “From 19,928 national participants, 9204 were excluded, yielding an analytic sample of 10,724 students. For Virginia, of 1954 participants, 88 were excluded, yielding an analytic sample of 1866 students” Missingness was higher in the national combined dataset because questionnaire content varies across participating sites, and some jurisdictions did not include all dietary intake items used to construct the FII. For multivariable regression, complete‐case analysis was used, excluding participants with missing covariate data.

CDC guidance for complex survey analysis was followed, including the application of sampling weights, strata, and primary sampling units to account for nonresponse and the complex survey design [32, 35]. Because the YRBSS datasets are publicly available and contain no personally identifiable information, our study is exempt from institutional review board review.

### 2.2 Primary Outcome: Sadness or Hopelessness

Our primary outcome was persistent sadness or hopelessness, assessed with the standard YRBSS item: “During the past 12 months, did you ever feel so sad or hopeless almost every day for 2 weeks or more in a row that you stopped doing some usual activities?” [31] Responses were coded as “Yes” or “No” to create a binary outcome variable. This measure is widely used as a proxy for adolescent depressive symptoms in public health surveillance [17, 28].

### 2.3 Exposure: FII

#### 2.3.1 Rationale and Conceptual Basis

The YRBSS includes an optional hunger item (“During the past 30 days, how often did you go hungry because there was not enough food in your home?”), which has served as the primary proxy for FI in prior research [17]. However, this hunger item was not included in Virginia's 2023 YRBSS, creating a measurement gap. While the Virginia 2023 survey did include the breakfast‐skipping question, relying on this single behavior alone offers a narrow view of FI, as it captures only one acute manifestation of scarcity. To address these limitations, we developed a composite FII that integrates multiple indicators of both acute and chronic dietary compromise, enabling consistent measurement across the Virginia and national datasets.

#### 2.3.2 Conceptual Basis

The FII was developed with reference to two complementary theoretical frameworks that together capture both immediate and long‐term behavioral responses to scarcity. The first is the Scarcity Adaptation Model, which describes how individuals adjust their daily routines under conditions of constraint [36]. When food is scarce, adolescents may adapt by delaying meals or skipping them altogether, behaviors most visibly reflected in breakfast skipping. Such short‐term coping strategies signal acute dimensions of FI [37, 38, 39].

The second is the ECLAIR model—Economic Constraints Leading to Altered Intake and Reduced Diet—which highlights how persistent economic pressures shape dietary trade‐offs [40]. Limited resources often drive families toward cheaper, calorie‐dense foods while reducing consumption of fruits, vegetables, and other nutrient‐rich options. Over time, these compromises reflect the chronic dimensions of FI, as nutritional quality erodes in the face of financial strain [41, 42, 43].

Taken together, these frameworks provide a strong theoretical justification for constructing an index that integrates indicators of both acute scarcity (e.g., meal skipping) and chronic dietary compromise (e.g., low fruit and vegetable intake, high sugary drink consumption, limited water intake). By aligning with both models, the FII is designed to capture the multidimensional character of adolescent FI rather than reducing it to a single behavior or experience.

#### 2.3.3 Indicator Selection

We identified five items that were available in both the Virginia and national 2023 YRBSS datasets to construct the composite FII:

- *Acute Food Insecurity*: Frequency of breakfast skipping (Q75).
- *Diet Quality*—Fruits and Vegetables: Frequency of fruit consumption (Q69) and green salad consumption (Q70).
- *Sugary Drink Consumption*: Frequency of drinking regular soda or pop (Q74).
- *Water Intake*: Frequency of drinking plain water (Qwater).

These indicators capture behavioral responses to limited food access that have been documented in FI research. When households face resource constraints, dietary consumption patterns often shift away from nutrient‐dense foods such as fruits and vegetables toward cheaper calorie‐dense options and sugar‐sweetened beverages. Adolescents may also skip meals—particularly breakfast—as a short‐term coping strategy when food availability is uncertain. Dietary behaviors captured in the YRBSS, including fruit and vegetable intake, sugary beverage consumption, and meal frequency, therefore serve as indirect indicators of nutritional compromise in adolescent populations.

By using only items present in both datasets, the FII provides a consistent and replicable measure of adolescent FI that goes beyond the single hunger or breakfast‐skipping items commonly used in prior studies.

#### 2.3.4 Validity Considerations

Previous YRBSS research has typically measured FI using a single proxy indicator—most commonly the optional hunger question or, when unavailable, breakfast skipping [17]. While useful, both measures have been critiqued for being overly narrow, as they fail to capture the broader dietary compromises that accompany constrained access to food [17]. The FII builds on this prior work by integrating multiple behaviors into a single composite, thereby reflecting both acute scarcity (meal skipping) and chronic compromise (low fruit and vegetable intake, high sugary drink consumption, reduced water intake). By combining these indicators, the FII enhances construct validity, provides consistency across the Virginia and national datasets, and offers a more nuanced surveillance tool than any single‐item proxy. The FII was therefore conceptualized as a surveillance‐oriented proxy measure designed to approximate FI in contexts where standard food security modules are unavailable, rather than as a validated psychometric scale.

The selection of these indicators was also guided by the practical constraints of the YRBSS dataset. Because the standard hunger question was not included in the 2023 Virginia YRBSS, it was necessary to approximate FI using available dietary and behavioral items that plausibly reflect coping responses to constrained food access. Rather than relying on a single behavior such as breakfast skipping, we combined multiple indicators to better approximate the multidimensional nature of FI within the constraints of this dataset. The FII should therefore be interpreted as a surveillance‐oriented proxy for FI, not a validated food security scale.

#### 2.3.5 Scoring System

Each item was coded on a scale from 0 (food secure) to 4 (high FI) based on its association with reduced diet quality or meal frequency. Because higher water intake is protective, we reverse‐coded the water‐consumption item so that lower intake received higher scores, ensuring that higher FII values uniformly indicate greater nutritional risk. However, it is also possible that increased water consumption may reflect a coping behavior when food is scarce—for example, adolescents drinking water to suppress hunger. Because the YRBSS does not capture contextual information about the reasons for beverage consumption, this alternative interpretation cannot be fully ruled out. We therefore interpret the water consumption indicator cautiously and discuss this potential ambiguity as a limitation of the composite index.

Item scores were summed to generate the FII, producing a total score ranging from 0 to 20. Categories of FI severity are illustrated in Table 1.

**TABLE 1:** Defining the food insecurity index (FII): Severity categories and score thresholds.

Score range	Category	Interpretation
0–4	Food secure	No reported food insecurity
5–8	Mild food insecurity	Some dietary limitations, but meals still regular
9–12	Moderate food insecurity	Reducing meal frequency; consuming less nutritious food
13–16	Severe food insecurity	Skipping meals regularly; unable to access sufficient food
17–20	Extreme food insecurity	Chronic food deprivation

*Note:* Cut points reflect both the observed distribution of scores and conceptual thresholds of increasing severity of dietary compromise. The FII was modeled as continuous in regression analyses; these categories are used only for descriptive purposes.

### 2.4 Covariates

Covariate selection was guided by prior YRBSS literature on adolescent behavioral health [17, 18, 44, 45]. We also adjusted for behavioral and school‐related risks. Bullying was measured as being bullied on school property in the past 12 months (Yes/No). Substance use included current cigarette smoking, vaping, alcohol use, and marijuana use (all coded Yes/No). School safety was assessed by whether students missed school due to feeling unsafe in the past 12 months (Yes/No). Physical activity was measured as engagement in ≥ 60 min of activity on at least 5 days in the past week (Yes/No). Because this study is cross‐sectional, our aim was not to identify causal pathways but to describe the association between FI and mental health while accounting for multiple overlapping risks. Some of these variables may themselves be influenced by FI, so the adjusted associations presented here should be interpreted as conservative.

### 2.5 Statistical Analysis

We first generated weighted descriptive statistics for both the national and Virginia samples, stratified by persistent sadness or hopelessness. Group differences were assessed using Rao–Scott chi‐squared tests for categorical variables and Mann–Whitney *U* tests for continuous variables, given their non‐normal distributions.

Cases with missing data on the outcome, predictors, or covariates were excluded using listwise deletion; no imputation was performed, and analyses were conducted on a complete‐case basis.

We then fit survey‐weighted logistic regression models to estimate the association between the FII and persistent sadness/hopelessness, adjusting for all specified covariates. Results are reported as adjusted odds ratios (AORs) with 95% confidence intervals (CIs). Statistical significance was defined as two‐sided *p* < 0.05.

To assess potential over‐adjustment from behavioral and school‐climate variables that may lie on the causal pathway between FI and mental health, we conducted nested sequential sensitivity analyses. Three nested models were estimated: Model 1 adjusted for demographic characteristics (age, sex, race/ethnicity); Model 2 additionally adjusted for physical activity; and Model 3 corresponded to the fully adjusted model including bullying, school safety, and substance use variables. These analyses evaluated whether the association between the FII and persistent sadness or hopelessness remained stable across alternative adjustment specifications.

All analyses incorporated CDC‐provided sampling weights, strata, and primary sampling units to account for the complex survey design. Analyses were conducted in R version 3.6.3 (R Foundation for Statistical Computing) using the survey package.

## 3 Results

### 3.1 Sample Characteristics

The analytic sample included 10,724 students nationally and 1866 students in Virginia. Median FII scores were 10 (IQR: 8–12) nationally and 9 (IQR: 7–11) in Virginia. Using categorical groupings, 16.1% of students nationally and 13.8% in Virginia experienced moderate or greater FI (Table 2). Higher FII scores were observed among students who were female, reported being bullied, engaged in vaping, or missed school due to feeling unsafe (all *p* < 0.001). In the national sample, Hispanic students had the highest median FII scores, followed by non‐Hispanic Black students (*p* < 0.001).

**TABLE 2:** Food insecurity measures by sadness or hopelessness status at the national and Virginia levels.

	National (*n* = 10,724)			Virginia (*n* = 1866)		
Characteristic	Feeling sad and hopeless (No)	Feeling sad and hopeless (Yes)	*p* ^a^	Feeling sad and hopeless (No)	Feeling sad and hopeless (Yes)	*p* ^a^
*N*	6041 (56.3%)	4683 (43.7%)		1242 (66.6%)	624 (33.4%)	
Median food insecurity index	10 (8–12)	11 (9–13)	< 0.001	9 (7–11)	10 (8–12)	< 0.001
Food insecurity category			< 0.001			< 0.001
Food secure (0–4)	261 (4.3%)	117 (2.5%)		84 (6.8%)	18 (2.9%)	
Mild food insecurity (5–8)	1652 (27.3%)	875 (18.7%)		462 (37.2%)	161 (25.8%)	
Moderate food insecurity (9–12)	2765 (45.8%)	2144 (45.8%)		513 (41.3%)	295 (47.3%)	
Severe food insecurity (13–16)	1254 (20.8%)	1397 (29.8%)		172 (13.8%)	139 (22.3%)	
Extreme food insecurity (17–20)	109 (1.8%)	150 (3.2%)		11 (0.9%)	11 (1.8%)	

*Note:* All values presented as unweighted *n* (%) for categorical variables and median (interquartile range) for continuous variables. Valid percentages are reported excluding missing counts. Weighted statistical tests are used to account for survey weights, and a *p*‐value < 0.05 was statistically significant.

^a^ Associated *p*‐value from a weighted Rao–Scott Chi‐squared test or Mann–Whitney *U* test.

### 3.2 Prevalence of Sadness or Hopelessness

Overall, 43.7% of students nationally and 33.4% in Virginia reported persistent sadness or hopelessness in the past year. Prevalence rose sharply with FI: among students with severe FI, 66.4% nationally and 58.7% in Virginia reported sadness/hopelessness, compared to 30.1% and 23.6%, respectively, among food‐secure peers (*p* < 0.001). This pattern demonstrates a clear dose–response gradient across FII categories (Table 2).

### 3.3 Multivariable Associations

In survey‐weighted logistic regression models (Table 3), FI remained significantly associated with persistent sadness or hopelessness in both datasets. Each one‐point increase in FII corresponded to 8.7% higher odds of poor mental health nationally (AOR = 1.087, 95% CI: 1.062–1.112, *p* < 0.001) and 6.6% higher odds in Virginia (AOR = 1.066, 95% CI: 1.005–1.130, *p* = 0.039).

**TABLE 3:** Adjusted odds ratios for factors associated with sadness or hopelessness at the national and Virginia levels.

Characteristic	National		Virginia	
	AOR (95% CI)	*p*	AOR (95% CI)	*p*
Food insecurity index	1.087 (1.062–1.112)	**< 0.001**	1.066 (1.005–1.130)	**0.039**
Age				
14 years old	*Reference*		*Reference*	
15 years old	0.938 (0.759–1.159)	0.548	1.700 (0.982–2.944)	0.055
16 years old	0.935 (0.735–1.191)	0.580	1.468 (0.875–2.460)	0.108
17 years old	0.999 (0.777–1.284)	0.991	1.405 (0.815–2.419)	0.157
18 years old and older	0.882 (0.689–1.129)	0.311	2.075 (0.925–4.657)	0.066
Sex (Female)	2.974 (2.618–3.378)	**< 0.001**	3.052 (1.977–4.711)	**0.002**
Race				
Non‐Hispanic White	*Reference*		*Reference*	
Non‐Hispanic Black	1.052 (0.857–1.292)	0.621	0.938 (0.640–1.376)	0.668
Hispanic	1.281 (1.063–1.544)	**0.010**	1.321 (0.835–2.091)	0.167
Other	1.189 (0.943–1.501)	0.140	1.305 (0.636–2.675)	0.362
Bullying	3.202 (2.698–3.800)	**< 0.001**	3.627 (1.882–6.991)	**0.006**
Current Cigarette Use	1.708 (0.973–2.996)	0.062	0.708 (0.132–3.789)	0.568
Current Vape Use	1.627 (1.269–2.085)	**< 0.001**	3.177 (1.997–5.053)	**0.002**
Current Alcohol Use	1.305 (1.118–1.523)	**0.001**	1.743 (0.988–3.076)	0.053
Current Marijuana Use	1.864 (1.490–2.332)	**< 0.001**	1.071 (0.565–2.032)	0.781
School Safety	2.037 (1.407–2.949)	**< 0.001**	1.740 (0.978–3.096)	0.056
Physical Activity	0.877 (0.736–1.043)	0.134	0.821 (0.541–1.244)	0.258

*Note:* AORs with 95% CIs from survey‐weighted logistic regressions of persistent sadness/hopelessness. FII modeled continuously (0–20; association per one‐point increase); national and Virginia models estimated separately. Two‐sided *p* < 0.05 was considered statistically significant. Bold values represent statistical significance and variables that meet the *p* < 0.05 threshold.

Abbreviations: AOR, adjusted odds ratio; CI, confidence interval.

The strongest correlates of poor mental health were female sex and bullying. Female students experienced nearly threefold higher odds of sadness/hopelessness compared to males (National AOR = 2.97, 95% CI: 2.62–3.38; Virginia AOR = 3.05, 95% CI: 1.98–4.71). Being bullied on school property tripled the odds in both datasets (National AOR = 3.20, 95% CI: 2.70–3.80; Virginia AOR = 3.63, 95% CI: 1.88–6.99).

Substance use was also associated with poor mental health, particularly vaping, which doubled or tripled the odds across samples (National AOR = 1.63, 95% CI: 1.27–2.09; Virginia AOR = 3.18, 95% CI: 1.99–5.05). Alcohol and marijuana use were significant in the national sample but not in Virginia.

Race/ethnicity was significant nationally, with Hispanic students having higher odds compared to non‐Hispanic White students (AOR = 1.28, 95% CI: 1.06–1.54). No significant racial/ethnic differences were observed in the Virginia sample. Other covariates, including cigarette use, school safety, and physical activity, were not consistently associated with mental health outcomes across models.

Sequential adjustment sensitivity analyses produced similar results (Table S1). In the national sample, the association between the FII and persistent sadness or hopelessness remained statistically significant across all specifications, though the magnitude attenuated slightly with additional adjustment (AOR = 1.113 in the demographic model, 1.109 after adding physical activity, and 1.087 in the fully adjusted model). A similar pattern was observed in the Virginia sample (AOR = 1.102, 1.098, and 1.066, respectively), indicating that the relationship between food insecurity and mental health remained robust across alternative model specifications.

## 4 Discussion

This study demonstrates a graded association between FI and persistent sadness or hopelessness among US and Virginia high school students. The relationship persists after adjustment for demographic, behavioral, and school‐climate covariates, and appears in both the national and state samples. Subgroup analyses indicate substantially higher odds of poor mental health among female students at comparable levels of FI, consistent with broader patterns of greater internalizing symptoms reported by adolescent girls [17, 46, 47]. Together, the findings suggest that nutritional scarcity is a salient correlate of adolescent mental health and that its burden is unevenly distributed across groups and geographies.

Two complementary mechanisms help interpret these results. First, under immediate constraints, adolescents may adopt short‐term coping strategies—most visibly breakfast skipping—that reflect acute scarcity and are linked to worse mood and concentration. Second, persistent economic pressures shift diets toward cheaper, energy‐dense, nutrient‐poor foods and away from fruits and vegetables, gradually eroding diet quality. These acute and chronic responses map onto stress physiology (heightened allostatic load), cognitive load (reduced bandwidth for self‐regulation), and social functioning (stigma, disengagement), each of which plausibly contributes to persistent sadness or hopelessness [48, 49].

Virginia provides a particularly informative setting. Youth mental‐health services are already stretched, and county data—more suicide‐risk screenings and more same‐day requests—show the need is rising. When systems are this strained, the mental‐health harm tied to FI is less likely to be eased by quick screening, referrals, or timely treatment. The replication of the FI–mental health association in Virginia therefore underscores the relevance of school‐based identification and linkage strategies where specialty care is comparatively scarce. Consistent with our study framing, the magnitude of association between FI and mental health outcomes in Virginia closely mirrors national patterns, suggesting that this relationship operates similarly even within a high‐need state context.

Our results align with prior work documenting stronger links between FI and adverse mental‐health outcomes in adolescents. The present study adds two contributions: (1) a multidimensional FII that captures both acute (meal timing/omission) and chronic (diet quality) scarcity signals when standard food‐insecurity modules are absent; and (2) a state–national comparison showing similar gradients even in a relatively lower‐prevalence state, suggesting that gradients—not just prevalence—carry policy significance. Importantly, these findings suggest that meaningful gradients in adolescent mental health can still be detected in surveillance datasets even when standard FI modules are unavailable. The FII also highlights the potential value of multidimensional proxy measures for surveillance research, particularly when standard food‐security modules are unavailable. Rather than relying on a single behavioral indicator, the index integrates several dietary behaviors that reflect both immediate coping responses to scarcity and longer‐term dietary compromises. This approach may provide a more nuanced picture of adolescent FI in school‐based surveillance systems such as the YRBSS, particularly in states where optional food‐security items are not included. Future research should further validate this approach by comparing composite indices such as the FII with established food‐security scales and examining their predictive value for health and educational outcomes.

Several analytic considerations merit comment. First, several behavioral and school‐climate variables (e.g., bullying, substance use, perceived safety) may lie on the causal pathway between FI and mental health. Because our question is policy‐facing—whether FI adds risk once schools account for problems they already observe—we emphasize fully adjusted models. These estimates likely bias toward the null if such variables partially mediate the association, but they provide the conservative, decision‐relevant quantity that schools can expect net of concurrent risks. Sensitivity analyses using sequential covariate adjustment produced similar patterns, with modest attenuation in association size but a consistently significant association between FI and persistent sadness or hopelessness (Table S1). Second, the FII uses equal weighting across components. We chose this for transparency and ease of implementation when standard food‐security items are unavailable; it avoids data‐driven overfitting and facilitates replication across jurisdictions. Future research should validate multidimensional proxy indices such as the FII against established food security screening instruments in adolescent populations. Third, although dietary items are self‐reported and subject to measurement error, such error typically attenuates associations toward the null rather than producing spurious links.

## 5 Limitations

This study is cross‐sectional; causality cannot be inferred, and reverse causation is possible. The FII is theory‐driven and useful in contexts where standard food‐security items are absent, but it is not a substitute for validated food‐security modules. Because the FII integrates several behavioral indicators rather than items measuring a single latent construct, it should be interpreted as a surveillance‐oriented proxy measure rather than a formally validated FI scale. All measures are self‐reported and may be misclassified; such error typically biases associations toward the null. We cannot observe out‐of‐school youth, and some contextual factors (e.g., neighborhood deprivation, local food prices, and wait times for counseling) are not measured.

Missing data were more substantial in the national sample due to variation in questionnaire content across jurisdictions, as not all sites included the dietary items used to construct the FII. Although complete‐case analysis and survey weights were applied, this differential missingness may introduce selection bias if excluded respondents differed systematically from those included. However, the consistency of findings across both the national and Virginia samples provides reassurance regarding the robustness of the observed associations.

Despite these limitations, the use of large, population‐based datasets and consistent patterns across independent samples and alignment with prior literature, strengthens confidence in the findings.

## 6 Implications for School Health Policy, Practice, and Equity

Policy and practice implications follow directly from these findings. For surveillance, the YRBSS program in Virginia should consider retaining the hunger optional item question in future surveys, and where modules vary, an index like the FII can preserve comparability and local decision utility. For school practice, pairing routine screening for food‐related risk with concrete supports—expansion of breakfast after the bell or grab‐and‐go options, reliable access to drinking water, school‐based pantries and produce distributions, and streamlined referrals—may help address both acute and chronic dimensions of scarcity [50, 51]. For behavioral health, schools and districts may benefit from strengthening care navigation and warm hand‐offs to community providers, prioritizing campuses with the highest FII burden. Finally, programming should be gender‐responsive and integrated with bullying prevention, given the consistently higher internalizing burden among girls and the salience of school climate [52, 53].

## 7 Conclusion

Across national and Virginia datasets, FI is consistently and monotonically associated with persistent sadness or hopelessness among high school students. These findings also demonstrate that multidimensional proxy measures derived from routine surveillance data may help identify meaningful gradients in adolescent FI when standard food‐security modules are unavailable. In a state with constrained youth behavioral‐health capacity, integrating food access supports with school‐based identification and care navigation may represent a feasible strategy to mitigate psychological risk. Consistent, brief surveillance of FI and targeted, gender‐responsive programming may therefore be important components of school health strategy.

Table S1 presents sequential adjustment sensitivity analyses examining the association between the Food Insecurity Index and persistent sadness or hopelessness across national and Virginia YRBSS samples. The table demonstrates the robustness of findings across progressively adjusted regression models.

## Conflicts of Interest

The authors declare no conflicts of interest.

## Supporting information


**Table S1:** Sequential adjustment sensitivity analysis for the association between the food insecurity index and persistent sadness or hopelessness‐National and Virginia YRBSS, 2023.

## Data Availability

Raw YRBSS data are publicly available from CDC. The scripts used to construct the FII and reproduce the analyses are available from the corresponding author upon reasonable request.
