# Supplementary material for: Food Insecurity and Mental Health Among High School Students: Evidence From National and Virginia Youth Risk Behavior Surveillance Data
Source: J Sch Health. 2026 Jun 8;96(7):e70172. doi: 10.1111/josh.70172 (PMC13244190; doi:10.1111/josh.70172)
Supplement: Supplementary file 1 — Table S1: Sequential adjustment sensitivity analysis for the association between the food insecurity index and persistent sadness or hopelessness‐National and Virginia YRBSS, 2023. [file JOSH-96-0-s001.docx]

Supplement Table S1. Sequential adjustment sensitivity analysis for the association between the food insecurity index and persistent sadness or hopelessness-National and Virginia YRBSS, 2023

National Sample

| **Models** | AOR | 95% CI | p-value |
| --- | --- | --- | --- |
| *Model 1 (Demographics only)* | 1.113 | (1.094-1.133) | <0.001 |
| *Model 2 (+ Physical activity)* | 1.109 | (1.086-1.132) | <0.001 |
| *Model 3 (Full Adjusted)* | 1.087 | (1.062-1.112) | <0.001 |

Virginia Sample

| **Models** | AOR | 95% CI | p-value |
| --- | --- | --- | --- |
| *Model 1 (Demographics only)* | 1.102 | (1.045-1.162) | 0.002 |
| *Model 2 (+ Physical activity)* | 1.098 | (1.041-1.157) | 0.003 |
| *Model 3 (Fully Adjusted)* | 1.066 | (1.005-1.130) | 0.039 |

*Notes:* Model 1 adjusted for age, sex, and race/ethnicity. Model 2 additionally adjusted for physical activity. Model 3 corresponds to the fully adjusted model including bullying, school safety, and substance use variables.
